# Supplementary material for: Empowering EFL teachers’ perceptions of generative AI-mediated self-professionalism
Source: PLoS One. 2025 Jun 23;20(6):e0326735. doi: 10.1371/journal.pone.0326735 (PMC12185028; doi:10.1371/journal.pone.0326735)
Supplement: Attachment — Submitted filename: Response_to_reviewers.docx [file pone.0326735.s002.docx]

PONE-D-24-28944

Empowering EFL Teachers’ Perceptions of Generative AI-Mediated Self-Professionalism

PLOS ONE

Dear Dr. Nazim,

Thank you for submitting your manuscript to PLOS ONE. After careful consideration, we feel that it has merit but does not fully meet PLOS ONE’s publication criteria as it currently stands. Therefore, we invite you to submit a revised version of the manuscript that addresses the points raised during the review process.

Please submit your revised manuscript by Nov 07 2024 11:59PM. If you will need more time than this to complete your revisions, please reply to this message or contact the journal office at [plosone@plos.org](mailto:plosone@plos.org). When you're ready to submit your revision, log on to https://www.editorialmanager.com/pone/ and select the 'Submissions Needing Revision' folder to locate your manuscript file.

Please include the following items when submitting your revised manuscript:

- A rebuttal letter that responds to each point raised by the academic editor and reviewer(s). You should upload this letter as a separate file labeled 'Response to Reviewers'.
- A marked-up copy of your manuscript that highlights changes made to the original version. You should upload this as a separate file labeled 'Revised Manuscript with Track Changes'.
- An unmarked version of your revised paper without tracked changes. You should upload this as a separate file labeled 'Manuscript'.

If you would like to make changes to your financial disclosure, please include your updated statement in your cover letter. Guidelines for resubmitting your figure files are available below the reviewer comments at the end of this letter.

If applicable, we recommend that you deposit your laboratory protocols in protocols.io to enhance the reproducibility of your results. Protocols.io assigns your protocol its own identifier (DOI) so that it can be cited independently in the future. For instructions see: [https://journals.plos.org/plosone/s/submission-guidelines#loc-laboratory-protocols](https://c05y1x9s.r.us-east-2.awstrack.me/L0/https:%2F%2Fjournals.plos.org%2Fplosone%2Fs%2Fsubmission-guidelines%23loc-laboratory-protocols/1/010f01921df7dad5-2e30fb03-66ef-4b31-be77-38e0a5672487-000000/i_T0jsNNjU7qq7eeXyFPglneClo=177). Additionally, PLOS ONE offers an option for publishing peer-reviewed Lab Protocol articles, which describe protocols hosted on protocols.io. Read more information on sharing protocols at [https://plos.org/protocols?utm_medium=editorial-email&utm_source=authorletters&utm_campaign=protocols](https://c05y1x9s.r.us-east-2.awstrack.me/L0/https:%2F%2Fplos.org%2Fprotocols%3Futm_medium=editorial-email%26utm_source=authorletters%26utm_campaign=protocols/1/010f01921df7dad5-2e30fb03-66ef-4b31-be77-38e0a5672487-000000/cDONmU2Tuj-xI1H7r0_UDxo-cX4=177).

We look forward to receiving your revised manuscript.

Kind regards,

Ömer Gökhan Ulum
Academic Editor
PLOS ONE

Journal requirements:

When submitting your revision, we need you to address these additional requirements.

1. Please ensure that your manuscript meets PLOS ONE's style requirements, including those for file naming. The PLOS ONE style templates can be found at

https://journals.plos.org/plosone/s/file?id=wjVg/PLOSOne_formatting_sample_main_body.pdf and

https://journals.plos.org/plosone/s/file?id=ba62/PLOSOne_formatting_sample_title_authors_affiliations.pdf

2. We note that you have indicated that there are restrictions to data sharing for this study. PLOS only allows data to be available upon request if there are legal or ethical restrictions on sharing data publicly. For more information on unacceptable data access restrictions, please see http://journals.plos.org/plosone/s/data-availability#loc-unacceptable-data-access-restrictions.

Before we proceed with your manuscript, please address the following prompts:

a) If there are ethical or legal restrictions on sharing a de-identified data set, please explain them in detail (e.g., data contain potentially identifying or sensitive patient information, data are owned by a third-party organization, etc.) and who has imposed them (e.g., a Research Ethics Committee or Institutional Review Board, etc.). Please also provide contact information for a data access committee, ethics committee, or other institutional body to which data requests may be sent.

b) If there are no restrictions, please upload the minimal anonymized data set necessary to replicate your study findings to a stable, public repository and provide us with the relevant URLs, DOIs, or accession numbers. For a list of recommended repositories, please see

https://journals.plos.org/plosone/s/recommended-repositories. You also have the option of uploading the data as Supporting Information files, but we would recommend depositing data directly to a data repository if possible.

We will update your Data Availability statement on your behalf to reflect the information you provide.

3. We note that you have referenced:

Atikler, A. (1997). The role of action research in self-development of an ELT teacher: A descriptive case study [Unpublished master's thesis] Bilkent University, Ankara.

Karaaslan, A.D. (2003). Teachers' perceptions of self-initiated professional development: A case study on Bakent University English Language teachers [Unpublished master thesis]. Middle East Technical University.

) which has currently not yet been accepted for publication. Please remove this from your References and amend this to state in the body of your manuscript: (ie “Bewick et al. [Unpublished]”) as detailed online in our guide for authors

http://journals.plos.org/plosone/s/submission-guidelines#loc-reference-style

Response: both references and in text citations have been removed.
[Note: HTML markup is below. Please do not edit.]

Reviewers' comments:

Reviewer's Responses to Questions

Comments to the Author

1. Is the manuscript technically sound, and do the data support the conclusions?

The manuscript must describe a technically sound piece of scientific research with data that supports the conclusions. Experiments must have been conducted rigorously, with appropriate controls, replication, and sample sizes. The conclusions must be drawn appropriately based on the data presented.

Reviewer #1: No

Reviewer #2: Partly

2. Has the statistical analysis been performed appropriately and rigorously?

Reviewer #1: No

Reviewer #2: Yes

3. Have the authors made all data underlying the findings in their manuscript fully available?

The PLOS Data policy requires authors to make all data underlying the findings described in their manuscript fully available without restriction, with rare exception (please refer to the Data Availability Statement in the manuscript PDF file). The data should be provided as part of the manuscript or its supporting information, or deposited to a public repository. For example, in addition to summary statistics, the data points behind means, medians and variance measures should be available. If there are restrictions on publicly sharing data—e.g. participant privacy or use of data from a third party—those must be specified.

Reviewer #1: Yes

Reviewer #2: No

4. Is the manuscript presented in an intelligible fashion and written in standard English?

PLOS ONE does not copyedit accepted manuscripts, so the language in submitted articles must be clear, correct, and unambiguous. Any typographical or grammatical errors should be corrected at revision, so please note any specific errors here.

Reviewer #1: Yes

Reviewer #2: No

5. Review Comments to the Author

Please use the space provided to explain your answers to the questions above. You may also include additional comments for the author, including concerns about dual publication, research ethics, or publication ethics. (Please upload your review as an attachment if it exceeds 20,000 characters)

Reviewer #1: The study addressed a timely topic. However, there are major issues with it. Please see my comments below:

- There are many sentences within the text needing relevant citations. Please fix this problem throughout the manuscript.

Response: The relevant citations have been added throughout manuscript.

- The gap the study is going to address needs to be more highlighted in the introduction section. Moreover, the significance of the study should be discussed for different stakeholders in this section.

Response: The gap has been highlighted in the introduction section. In addition, the significance of the study has been discussed for different stakeholders in this section. (Introduction pages3-7)

- If the authors used a researcher-made questionnaire to gain the data, how did they measure the construct validity of the questionnaire?

Response: The researchers used a researcher-made questionnaire and the detailed have been provided. (page 16)

Response: A detailed information on how the researchers measured the construct validity of the questionnaire has been provided. (pages 19-22)

- The all steps to gather the data need to be reported in details. For example, it should be reported how the authors conducted the semi-structured interview.

Response: The steps to gather the data has been reported in detail under the data collection section. The procedure for conducting the semi-structured interview has been reported in detail. (page 16)

- More information about the data analysis should be provided. Unfortunately, the procedures reported to analyze the qualitative data are vague. For example, it is not clear how the authors measured the reliability and validity of the obtained findings.

Response: More information about the qualitative data analysis has been provided. The procedures to measure the reliability and validity of the obtained findings have also been reported. (pages 27-28)

- The required information about the tables need to be added. Moreover, please make sure the tables are set in line with the guidelines of APA 7the edition.

Response: The required information about the tables has been added in line with the guidelines of APA 7the edition.

- The qualitative results need to be presented in a common way in which the required analysis among the excerpts should be added to the text.

Response: The qualitative findings along with an analysis and interpretation of the selected excerpts have been added as required. (pages 32-36)

- The discussion part is not critical. In line with the literature, the authors should be justified the obtained findings in tune with the literature. Moreover, please discuss the theoretical and practical implications of the results in this part.

Response: The discussion section has been revised with a critical analysis that aligns with the literature review, and the findings are supported by relevant citations. In addition, the theoretical and practical implications of the results have been added to this part. (Discussion pages 36-45)

- The implications of the findings should be discussed by giving tangible examples how they can be used by different stakeholders.

Response: The implications of the findings have been discussed with examples along with an illustration on how they can be used by different stakeholders. (page 45)

- Given the limitations imposed on the study further suggestions for future study should be illuminated.

Response: The suggestions for future study have been revised. (pages 47)

- The language of the manuscript should be improved by doing more comprehensive proofreading.

Response: A comprehensive proofreading of the manuscript has been done, and the language has been improved for clarity and coherence.

Reviewer #2:

1. In the introduction section, it is recommended to highlight the innovative aspects of this study to emphasize its unique contributions to the field.

Response: In the introduction section, the innovative aspects of this study to emphasize its unique contributions to the field has been revised and highlighted. (Introduction pages3-7)

2. In the second part of the literature review, when discussing the relationship between teachers and technology, many statements lack supporting literature or cite outdated sources. It is advised to incorporate recent studies, such as:

Response: In the second part of the literature review, additional/recent studies have been incorporated, addressing the reviewer’s suggestions. (pages 9-11)

3. In the questionnaire section, it is recommended to detail which original questionnaires were utilized and specify any modifications made based on the requirements of this study.

Response: The details of the questionnaire developed by the researchers are provided, including insights from referenced original questionnaires (pages 16-17).

4. The content related to semi-structured interviews should be further expanded to provide a more comprehensive understanding of the interview process and findings.

Response: The steps related to semi-structured interviews have been reported in detail. (page 18)

Response: The analysis and interpretation of the interview findings have been added under the relevant themes. (pages 32-36)

5. It is suggested to add a dedicated section on data collection to clearly outline the procedures and methodologies employed in gathering data.

Response: A dedicated section on data collection has been added to clearly outline the procedures and methodologies employed in gathering data. (page 16)

6. In the data analysis section, it is recommended to merge single sentences into cohesive paragraphs to enhance the clarity and flow of the analysis process.

Response: In the data analysis section, single sentences are merged into a cohesive paragraph to enhance the clarity and flow of the analysis process. (page 26)

7. In the research findings section, the amount of interview data presented under each dimension is excessive. It is advised to consolidate these interviews or distribute them across more dimensions for better organization and readability.

Response: In the research findings section, the excerpts from the interviews have been revised and distributed under sub-themes to enhance organization and readability. (pages 32-36)

8. The discussion should be organized around the research questions and include relevant subheadings to ensure logical coherence and clarity in the analysis.

Response: The discussion section has been organized around the research questions and relevant subheadings are added for logical coherence and clarity. (Discussion pages 36-45)

9. The final paragraph of the conclusion discusses pedagogical implications, which is also addressed in the last paragraph of the discussion section. It is recommended to merge these sections to avoid redundancy and streamline the presentation of pedagogical insights.

Response: The sections have been merged to eliminate redundancy, and the final paragraph of the conclusion now includes recommendations and pedagogical insights. (page 48)

10. It is advised to limit each paragraph to a manageable number of words to improve the overall readability and maintain the reader’s engagement throughout the article.

Response: Each paragraph has been revised and limited to a manageable number of words to improve the overall readability and maintain the reader’s engagement throughout the manuscript.

6. PLOS authors have the option to publish the peer review history of their article (what does this mean?). If published, this will include your full peer review and any attached files.

If you choose “no”, your identity will remain anonymous but your review may still be made public.

Do you want your identity to be public for this peer review? For information about this choice, including consent withdrawal, please see our Privacy Policy.

Reviewer #1: No

Reviewer #2: No
