# Supplementary material for: Empowering EFL teachers’ perceptions of generative AI-mediated self-professionalism
Source: PLoS One. 2025 Jun 23;20(6):e0326735. doi: 10.1371/journal.pone.0326735 (PMC12185028; doi:10.1371/journal.pone.0326735)
Supplement: Attachment — Submitted filename: Response_to_Reviewers_auresp_2.docx [file pone.0326735.s003.docx]

**Reviewer #1:** Thank you for allowing me to review your manuscript. After careful consideration, I regret to inform you that I cannot recommend this paper for publication in its current form. The study suffers from significant methodological flaws and lacks the rigor required for publication in this journal. Please see my comments below:
1. The research questions suggest the need for a mixed-methods approach, as they aim to explore both quantitative and qualitative dimensions of the phenomenon under investigation. However, the study design does not reflect this requirement. The authors have not adequately justified their choice of methodology, nor have they demonstrated how the chosen design aligns with the research objectives. Furthermore, the methodology section is cluttered with irrelevant and redundant information, which detracts from the clarity and coherence of the study. A well-structured methodology section should clearly outline the research design, justify its appropriateness, and avoid unnecessary details.
Response

The methodology section has been revised. It aligns with the research objectives and appropriately justifies the chosen research design. It now provides a clear rationale for the methodology, demonstrating how it supports the research questions. Additionally, redundant and irrelevant details have been removed to enhance clarity and coherence.

2. The participants’ section is disorganized and lacks critical information. It should focus on key elements such as the demographic characteristics of the participants, the sampling method (e.g., random sampling, purposive sampling), inclusion and exclusion criteria, and the process of obtaining informed consent. Instead, the current section is filled with extraneous details that do not contribute to understanding the study population. Additionally, the authors have not clarified how they recruited participants or ensured ethical considerations, such as confidentiality and voluntary participation. These omissions raise concerns about the transparency and reproducibility of the study.

Response

The participants’ section has been revised to ensure clarity and completeness. The section now includes detailed information on the demographic characteristics of the participants, the sampling method employed, as well as the inclusion and exclusion criteria. Additionally, the recruitment process and the ethical considerations undertaken have been outlined, which include confidentiality measures and voluntary participation.

3. The authors claim to have used a researcher-made questionnaire but fail to provide a theoretical foundation for its design. A well-constructed questionnaire should be grounded in established theories or frameworks, and its development process should be clearly documented. Moreover, the authors have not addressed the construct validity of the questionnaire, which is typically assessed through exploratory factor analysis (EFA) and confirmatory factor analysis (CFA). Without evidence of construct validity, the credibility of the quantitative findings is severely compromised. This is a major flaw that undermines the scientific rigor of the study.
Response

Keeping the importance of grounding the questionnaire in established theories and ensuring its validity, a theoretical foundation (TPCK framework) has been added. Additionally, a detailed explanation of the development process has also been provided. Furthermore, regarding construct validity, exploratory factor analysis (EFA) and results have been added.

4. The qualitative data analysis section is inadequately described. The authors have not specified which data analysis method (e.g., thematic analysis, grounded theory, content analysis) was used, nor have they provided a clear step-by-step account of the analysis process. Additionally, there is no discussion of how the reliability and validity of the qualitative findings were ensured. For example, the authors could have employed strategies such as triangulation, member checking, or inter-coder reliability to enhance the trustworthiness of their results. The lack of transparency in this section makes it difficult to assess the quality of the qualitative findings.

Response

A description of the qualitative data analysis procedure has been provided, along with a step-by-step outline of the thematic analysis. Additionally, all strategies employed to ensure the reliability and validity of the qualitative findings have been added.

5. The presentation of the qualitative results is subpar and does not meet the standards expected in high-quality journals. The findings are not organized in a coherent manner, and there is insufficient integration of direct quotes or illustrative examples to support the authors' interpretations. I recommend that the authors consult established journals in their field to learn how to effectively present qualitative findings, including the use of thematic frameworks, tables, and narrative explanations.

Response

The presentation of the qualitative results has been revised to enhance clarity, and coherence. The findings are now structured using a thematic framework, with improved organization and integration of direct quotes and illustrative examples to support the interpretations. Additionally, tables and narrative explanations have been incorporated

6. The discussion section fails to adequately contextualize the findings within the existing literature. The authors have not sufficiently explained how their results align with or diverge from previous studies, nor have they addressed the theoretical and practical implications of their findings. A strong discussion should critically engage with the literature, highlight the contributions of the study, and acknowledge its limitations. The current discussion is superficial and does not provide a meaningful synthesis of the results.

Response

The discussion section has been revised to better contextualize the findings of the current study within the existing literature. The analysis has been expanded to compare the current study’s results with previous research, highlighting both similarities and differences. Additionally, the theoretical and practical implications of the findings have been elaborated to ensure a more comprehensive discussion. Moreover, a dedicated section on the study’s contributions has been added, while the limitations have been acknowledged and refined in the conclusion section.

**--------------------------------------------------------------------------------------------------------------------**

**Reviewer #2:** The article has significantly improved after revisions, with only a few areas requiring further modification. A minor revision is recommended. Specific suggestions are as follows:
1. In the abstract, it is recommended to include the data analysis methods to provide readers with a clearer understanding of the study's approach.

Response

The abstract has been revised, and the data analysis methods have been incorporated

2. In the last paragraph of the introduction, it is suggested that the author state the overarching objective of the study first, and then naturally lead into the research questions.

Response

In the last paragraph of the introduction, the objectives of the study have been added.

3. In the “Population and Sample” section, provide details on the inclusion criteria for participants. Additionally, the current single paragraph is overly lengthy—split it into two paragraphs for better readability and clarity.

Response

In the “Population and Sample” section, the inclusion criteria for participants have been provided.

4. In the conclusion, the final paragraph is redundant as it repeats earlier statements. It is recommended to delete this paragraph to avoid repetition.

Response

In the conclusion section, the final paragraph has been deleted.

**---------------------------------------------------------------------------------------------------------------------**

**Reviewer #3:** The paper is very interesting, yet the following are to be considered:
1. Editing is recommended.

Response

The manuscript has been edited.

2. The word ‘discovering’ at the beginning of the third question is better changed to ‘finding out’.

Response

The word ‘discovering’ at the beginning of the third question is changed to ‘finding out’.

3. Table 1 should immediately come after the reference on page 13.

Response

Table 1 has been placed right after its reference.

4. Table 1 is messy and confusing. It is better to expand it so the details appear separated.

Response

Table 1 has been expanded.

5. No need for the definite article ‘The’ in the titles of tables or figures.

Response

The definite article ‘The’ in the titles of tables has been deleted.

6. Table 2 is not situated under the sentence that stated this point on p.21. On p. 22, there is another reference to Table 2.

Response

Table 2 (now table 4) has been fixed.

7. I think the researchers mean ‘internal’ rather than ‘international’ in “Pearson’s coefficients were applied to check the international consistency of the”.

Response

The word ‘international’ has been replaced with ‘internal’.

8. Table 2 shows that only 10 respondents to the first domain and 20 to the second domain while the researchers’ statement in the previous page does not make this point clear. Fix this issue, please.

Response

Please note that the table 2 (now table 4) presents 10 items/statements under the first domain and 20 items/statements to the second domain and not the participants.

9. Sections in the Results section should be numbered to align with the pre-set research questions.

Response

The sections in the ‘Results’ have been numbered to align with the research questions.

10.The constrains and solutions in the Results sections need to appear in terms of recurrent themes.

Response

The constraints and solutions in the Results section are organized into separate sections, as they correspond to two distinct research questions.

11. The conclusion section should be divided into four sections as replies to the research questions.

Response

To the researchers’ mind, this comment pertains to the ‘Discussion’ section, which has already been structured into sections corresponding to the research questions.

12.Do not use the ampersand ‘&’ within the text as you have in “Gill et al. (2024) and Samek & Müller (2019) indicate that”. Use it only within the in-text citation.

Response

This issue has been fixed.

13.There is some redundancy in some sections including the Conclusion section.

Response

The manuscript has been revised to eliminate all instances of redundancy, ensuring clarity and conciseness. This includes revising the Conclusion section to enhance coherence and avoid repetition. All necessary adjustments have been made to improve the overall flow and readability of the manuscript.

14.The list of references is to be reconsidered due to having some inconsistency in some references.

Response

The references section has been updated.
