# Supplementary material for: Empowering EFL teachers’ perceptions of generative AI-mediated self-professionalism
Source: PLoS One. 2025 Jun 23;20(6):e0326735. doi: 10.1371/journal.pone.0326735 (PMC12185028; doi:10.1371/journal.pone.0326735)
Supplement: Attachment — Submitted filename: Response_to_Reviewers_auresp_4.docx [file pone.0326735.s005.docx]

Reviewer #1:

This manuscript has improved significantly after one round of revisions, with enhanced clarity, methodology, and structure. However, certain areas still require further refinement. Specific suggestions are outlined below.

1. In the first two paragraphs of the introduction, it is recommended to include more references from the past five years.

Response

More references from the past five years, in the first two paragraphs of the introduction, have been included. (Pages 3-4)

2. In the introduction, the last three paragraphs should be merged and concisely focus on the research design, innovations, and significance of this study.

Response

In the introduction, the last three paragraphs have been merged with a focus on the research design, innovations, and significance of this study. (pages 6-7)

3. In the literature review section, when discussing the application of AI in university language education, it is recommended to include some recent studies.

Response

In the literature review, while discussing the application of AI in university language education, more recent studies have been included. (page 10)

4. In the "Research Design and Context" section, it is recommended to remove the statement about the significance of the study at the end of the paragraph. This section should focus solely on the research design and context, as the significance of the study has already been addressed at the end of the introduction.

Response

In the "Research Design and Context" section, the statement about the significance of the study at the end of the paragraph has been removed. (page 13)

5. In the methodology section, it is suggested to follow the logic of "research design - participants – instruments - data collection - data analysis."

Response

In the methodology section, the logic of "research design i.e. participants – instruments - data collection - data analysis has been followed. (pages 13-28)

6. The presentation of the conclusion section has some issues. It is recommended to structure it according to three dimensions: summarizing the research findings, identifying research limitations, and suggesting future research directions.

Response

The presentation of the conclusion section has been structured according to three dimensions: summary of the research findings, research limitations, and suggestions for future research directions. (pages 52-54)

Reviewer #2:

Dear author,
Thanks for addressing most of the comments left, yet some other issues need to fix:
1. Editing is still needed.

Response

The manuscript has been revised and edited as needed.

2. The four objectives should appear in the -ing form (you have three infinitive and one gerund).

Response

The four objectives have been revised and now they appear in the -ing form. (page 8)

3. Since the figure or table are identified by the number, there is no need for the definite article. 'the' as in "The figure (1) and table (2)". The same mistake is repeated in other places. Fix all, please.

Response

Figures and tables have been revised, and all occurrences of the definite article ‘the’ have been removed and fixed. (page 18-21)

4.Still there is no consistency in writing the sources in the reference list. When there is an article from a journal, a colon is used after the volume and issue followed by the beginning and ending of the pages. Check all, please.

Response

All references have been checked, and colons have been replaced with commas.

5. Some titles are not italicized in the reference list.

Response

All references have been checked, and the titles have been italicized.
